# Intra- and Extracranial MR Venography: Technical Notes, Clinical Application, and Imaging Development

**DOI:** 10.1155/2016/2694504

**Published:** 2016-05-31

**Authors:** M. Paoletti, G. Germani, R. De Icco, C. Asteggiano, P. Zamboni, S. Bastianello

**Affiliations:** ^1^Institute of Radiology, University of Pavia, Viale Golgi 19, 27100 Pavia, Italy; ^2^Department of Neuroradiology, IRCCS Istituto Neurologico Casimiro Mondino, Via Mondino 2, Pavia, Italy; ^3^Department of Brain and Behavioral Sciences, University of Pavia, Via Bassi 21, Pavia, Italy; ^4^Unit of Translational Surgery, University of Ferrara, Via Aldo Moro 8, Ferrara, Italy

## Abstract

Scientific debate over chronic cerebrospinal venous insufficiency (CCSVI) has drawn attention to venous system involvement in a series of pathologic brain conditions. In the last few decades, the MRI venography (MRV) field has developed a number of valuable sequences to better investigate structural anatomy, vessel patency, and flow characteristics of venous drainage in the intra- and extracranial systems. A brief two-tier protocol is proposed to encompass the study of intra- and extracranial venous drainage with and without contrast administration, respectively. Contrast-enhanced protocol is based on time-resolved contrast-enhanced MRV of the whole region plus extracranial flow quantification through 2D Cine phase contrast (PC); non-contrast-enhanced protocol includes intracranial 3D PC, extracranial 2D time of flight (TOF), and 2D Cine PC flow quantification. Total scanning time is reasonable for clinical applications: approximately seven minutes is allocated for the contrast protocol (most of which is due to 2D Cine PC), while the noncontrast protocol accounts for around twenty minutes. We believe that a short though exhaustive MRI scan of the whole intra- and extracranial venous drainage system can be valuable for a variety of pathologic conditions, given the possible venous implication in several neurological conditions.

## 1. Introduction

Over the last several years, attention has been increasingly dedicated to noninvasive imaging of the intra- and extracranial venous drainage systems [1, 2], especially following debate in the scientific community over chronic cerebrospinal venous insufficiency (CCSVI), a condition initially associated with multiple sclerosis (MS) [3]. Several articles have been published both in support of and against the initial hypothesis of a venous pathologic involvement in MS, drawing attention to the intra- and extracranial venous systems that, previously, had not been widely investigated.

Digital subtraction angiography (DSA) is traditionally considered the gold standard for venous studies, but the invasiveness of the procedure restricts its use to the time of treatment, while in clinical practice Doppler Sonography (DS) has progressively become the first-line exam for extracranial vessel study due to its noninvasiveness and practical clinical feasibility. As in any other ecotomographic study, however, DS studies of the internal jugular vein (IJV) system have well-known inter- and intraoperator reproducibility issues and are particularly influenced by patient compliance, respiration, postural changes, cardiac function, and adjacent arterial pulsations.

In contrast to shortcomings of these other methods, MRI and MR venography (MRV) of the neck gained great acceptance in the medical community due to their noninvasiveness, high reproducibility, lack of radiation exposure, field of view, and the possibility of investigating the venous system as a whole.

Consequently, MRI and MRV assumed an increasingly important role in detection of extracranial venous abnormalities, augmenting or replacing DS imaging in spite of their higher cost.

This added to the role of MRI, which was already heavily relied upon in the study of intracranial venous drainage using 2D and 3D time of flight (TOF) and phase contrast (PC) sequences for noncontrast imaging [4, 5] and contrast-enhanced MRV (CE-MRV) [6].

In recent years, MRI and MRV were considered for the assessment of hemodynamic characteristics of the jugular veins system in patients with multiple sclerosis suspected of having CCSVI. Several technical recommendations and protocols for venous system MRI study have been provided with a detailed description of* pros* and* cons* of all imaging sequences currently used [7–11]. Zivadinov et al. proposed an even wider evaluation of the venous system, encompassing not only intra- and extracranial venous drainage systems but also an assessment of venous anatomy and flow in the azygos system [12].

Possible implication of venous system abnormalities has been posited in a number of neurological diseases, such as primary exertional headache, cough headache and transient global amnesia [13–18]. Associations have been sought between white matter abnormalities and jugular flow reflux in mild cognitive impairment and Alzheimer disease. Although these are just pilot studies, reflux in the jugular system is generally considered a negative prognostic factor in the disease progression [19, 20].

Parallel to the growing debate over the venous system, the presence of a functional lymphatic system in the central nervous system, which has been recently posited in mice by Louveau et al., has drawn attention to traditional dogma regarding immune privilege of the brain. If confirmed in humans, this would mean a complete reformation of theories regarding the lymphatic and venous systems' role in pathology, especially for neuroimmunological diseases [21].

## 2. Venous Drainage Abnormalities Study

A number of different anatomical and pathologic conditions can influence and determine abnormalities in venous drainage. Due to this complexity, often only a multimodal approach can succeed in providing a final diagnosis. A combination of MRI and MRV sequences may be a successful approach to study different types of venous pathologies or paraphysiological conditions that could alter blood drainage.

Following a previous classification of venous drainage abnormalities by Zivadinov et al. [13], both intra- and extracranial abnormalities can be divided into extraluminal and intraluminal conditions.

Vessel narrowing (defined as reduction of vessel caliber ≥ 50%) and annulus are the most common extraluminal conditions. Conventional MRI with standard T1w and T2w sequences represents the diagnostic basis for any mass effect from adjacent structures on venous vessels. Conventional imaging and MRV (contrast-enhanced and non-contrast-enhanced) are together fundamental to evaluate the whole course of the vessel, its patency, and any eventual developmental abnormality or congenital caliber reduction and to show focal wall thickenings that may represent annuluses, as well. Even anatomical conditions like arachnoid granulations may have mass effect on venous vessels, so conventional MRI still has a key role in their diagnosis.

Intraluminal conditions encompass abnormalities of the internal wall of the vessel including abnormal valves, webs, septa, and flaps. Such pathologic conditions are generally hard to detect with MR imaging, as they may stand below the spatial resolution of venography sequences. As a result, these conditions may only be indirectly shown as a “minus image” after contrast administration with CE-MRV or as flow reduction with flow-dependent sequences. For a detailed evaluation of such conditions, DS and DSA remain fundamental.

Flow abnormalities are usually studied using DS imaging; the possibility of performing dedicated maneuvers to elicit specific flow characteristics in certain conditions is a valuable diagnostic tool in these cases. This level of detail is not achievable with MR imaging, though 2D Phase Contrast Imaging is able to show flow characteristics and eventual refluxes through a selected section of the vessel. 2D Phase Contrast Imaging provides flow information that is not operator-dependent and more reproducible than any DS measurement. In addition to this, 2D and 3D flow-dependent sequences are able to indirectly show vessels or segments of vessels with reduced flow. This panoramic view undoubtedly informs the whole diagnostic interpretation.

Collateral drainage is efficiently and promptly shown both with non-contrast-enhanced and with contrast-enhanced MRV sequences. Furthermore, CE time-resolved imaging is intrinsically designed to show temporal timing of venous blood return via its continuous “angiographic-like” acquisition of images, providing useful information about flow characteristics over time.

MRI and MRV are generally used for diagnosing venous thrombotic pathology, at both acute-subacute and chronic stages. Venography sequences are used to display characteristics of flow and structural imaging is used to characterize the temporal stage of hematic deposits, according to T1 and T2^*∗*^ relaxation times. MRI and MRV are also the primary radiological exam choice for diagnosis of idiopathic intracranial hypertension (IIH) and intracranial hypotension [6].

All things considered, MRV study efficiently enables a wide overview of the entire intra- and extracranial venous drainage systems in a relatively short time (especially with contrast-enhanced imaging), providing information that would otherwise have to be collected separately and then integrated.

Our opinion is that a simple and short (though quite exhaustive) MRV protocol for intra- and extracranial venous system study may be useful in clinical daily life to better investigate suspected structural or flow venous abnormalities that can be linked to a series of neurological and upper-spine diseases. We propose the use of this protocol in addition to traditional structural T1w and T2w sequences and alternatively or complementarily to DS studies due to its diagnostic reliability, noninvasiveness, and high level of reproducibility.

We adopted and refined a two-tier MRV study protocol, one including intravenous contrast administration and the other including only non-contrast-enhanced sequences. Each sequence presents some* pros* and* cons* that will be reported and discussed in the following paragraphs.

Complete intra- and extracranial MRV protocol are required to assess the venous drainage system as a whole. This protocol should have a wide field of view that permits an evaluation of eventual relationships with adjacent anatomical or pathologic structures and visualization of the whole course of vessels and collaterals (if present) and be able to quantify flow characteristics at certain standard levels for IJVs and at further levels, if needed.

Our proposed protocol encompasses the main widely available MRV techniques, including both non-contrast-enhanced MRV and CE-MRV; it does not, however, include advanced techniques that may be not easily accessible or easily read or that would still require wide acceptance and validation by the scientific community.

In our opinion, availability of non-contrast-enhanced venography sequences that rely on flow and that have been validated is, undoubtedly, helpful for a number of clinical situations in which the patient cannot undergo contrast administration.

## 3. Equipment and Technical Parameters

Current proposed MRV protocol has been performed and refined using a 1.5T scanner (Philips Achieva) equipped with a 16-channel receiving coil (Philips SENSE NV), located at Department of Neuroradiology, IRCCS Istituto Neurologico Mondino, Pavia, Italy. No structural MRI sequences are included in the protocol, as we focus specifically on venographic sequences.

In the extracranial venous drainage system, both protocols provide qualitative and quantitative imaging of the IJV system, giving the opportunity not only to study anatomic vessel abnormalities but also to quantify blood flow at specific levels of the IJV system.

Contrast administration necessary for time-resolved MRV is performed by an automatic contrast-injector (MEDRAD Spectris MR Injector SHS 200) with a single intravenous dose (0.01 mmol/kg) of gadobutrol (Gadovist 1.0, Schering AG, Switzerland), at a 1.5 mL/s rate initiated simultaneously with the start of the angiography sequence, followed by a 20 mL bolus of saline at the same rate.

Scanning time is less than seven minutes for the contrast protocol, most of which is due to 2D Cine PC, and twenty minutes for the full noncontrast protocol. These scan times are reasonable for an evaluation of the whole intra- and extracranial venous drainage. Proposed two-tier protocol is reported in Table 1.

### 3.1. Intracranial 3D Phase Contrast (PC) MRV

Phase contrast MR venography, which uses velocity-induced phase shifts to show flowing blood [22, 23], is typically used to perform MR venography with contrast administration being unnecessary. PC MRV is based on a GRE sequence to which a bipolar velocity-encoding gradient pulse and a velocity-encoding variable, known as VENC (which applies to the bipolar gradient strength to produce a phase shift of 180°), are added to encode blood velocity.

First, one gradient echo (GRE) sequence is acquired without flow encoding while other datasets are acquired with the bipolar gradients applied along the *x*-, *y*-, and *z*-axes. The intracranial venous system is then visualized through a combination of acquisitions sensitive to multioriented flows, and MIPs are computed to produce an “angiographic-like” image [6].

Recognized major advantages of PC MRV include optimized suppression of stationary background tissues (greater than TOF imaging) together with the ability to quantify flow and determine flow direction. Furthermore, detection of slow flows (for which PC MRV is superior to TOF imaging) can be improved by using a small voxel size, thus obtaining a better definition of small vessels and potential pathologic venous structures [24].

As a result of PC MRV's ability to detect slow flows, vascular lumens may appear larger than they do in TOF imaging because the periphery of vessels may saturate and then become invisible due to slow-flowing blood along vessel walls [25].

Finally, PC imaging enables a clear distinction between blood flow signal and an eventual thrombus, both of which have high signal on TOF imaging, leading to possible misdiagnosis.

PC imaging has several disadvantages to be considered. Its relatively long acquisition, due to the fact that three gradient directions have to be acquired to detect all possible flow directions, can lead to greater motion artifact susceptibility. This long acquisition time, however, can be reduced using parallel imaging.

Secondly, PC imaging requires that the optimal VENC, which cannot be precisely known in advance, is predicted* a priori*. This complicates the successful execution of the technique, making PC imaging more operator-dependent. For these reasons, 2D PC MRV technique, which uses thick slab images, is sometimes employed. This method, however, can produce unconvincing results, even in MIP reconstructions.

Other options for intracranial venous evaluation are offered by 2D and 3D TOF MRV. Liauw et al. did not recommend intracranial 3D TOF MRV because of severe in-plane saturation, preferring 2D TOF visualized as MIP reconstructions or 3D PC MRV for an overall better quality [4]. For years, these two sequences have been generally considered comparable for non-contrast-enhanced detection of intracranial venous structures [6], although PC imaging has been less used for long acquisition times.

In recent years, availability of a 3D PC MR angiography (MRA) sequence that employs parallel imaging and optimized *k*-space sampling enabled a high resolution angiographic acquisition with excellent background suppression and a considerably shortened scan time [14, 26].

**Figure 1:**
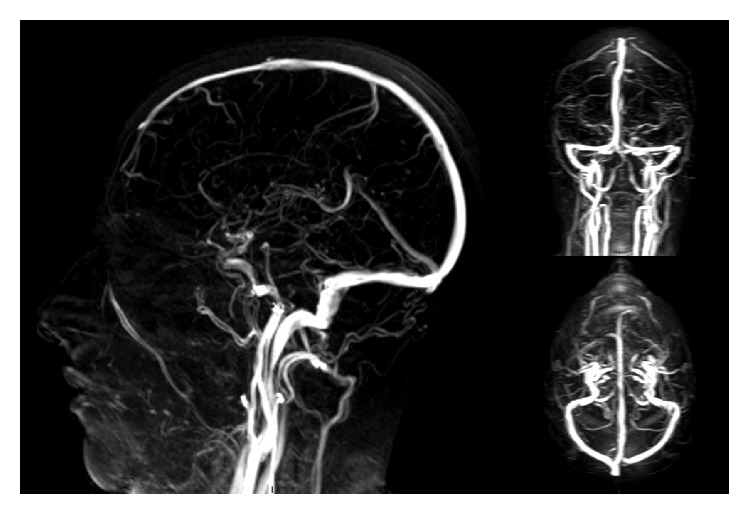
MIP sagittal, coronal, and axial reconstructions of intracranial 3D phase contrast venography.

Our belief is that 3D PC MRV with the most recent technical adjustments represents a great diagnostic opportunity for patients who cannot undergo contrast administration, enabling high-quality study of the intracranial venous system that, quite often, proves to be not inferior to contrast-enhanced techniques [26] (Figure 1).

### 3.2. Extracranial 2D TOF MRV

TOF MR angiography is based on the principle that blood flowing into an imaging section has higher signal intensity than the stationary tissue within that section, which is partially saturated.

An arterial or venous phase is possible according to clinical needs: if a venous study of the neck is performed, any flow toward the head (i.e., arterial flow) will be discarded using a saturation band and the flow toward the heart (i.e., venous flow) will be highlighted in a velocity-dependent manner. In a reasonably short acquisition time, TOF imaging provides a good overview of the venous system, allowing an evaluation of structural vascular anatomy and vessel patency. In addition to this, if high resolution data are collected, vessel cross section can be calculated to evaluate the degree of caliber reduction.

In 2D TOF, a series of thin slices is collected in the axial plane perpendicular to the jugular axes, covering the extracranial region from the aortic arch upwards to at least transverse sinuses.

**Figure 2:**
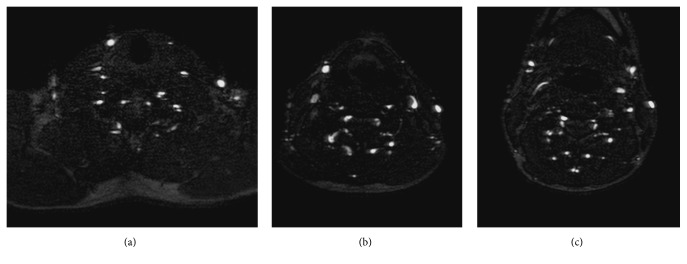
Extracranial 2D TOF: axial slices at different neck levels, (a) at the neck basis, (b) at midneck level (C5-C6 level), and (c) at an immediately submandibular level (C4-C5 level), showing patency of the IJV system bilaterally, as that of anterior jugular veins (with right side prominence) and vertebral venous drainage.

As with other venographic imaging techniques, MIP reconstructions are commonly used but, because of the high number of neck vessels and well-known precautions that have to be adopted to correctly interpret MIPs, axial images are often extremely valuable (Figure 2).

Limitations to this technique are mainly related to artifacts resulting from slow blood flow, in-plane flows that are usually saturated, and turbulent and/or pulsatile flow patterns [27]. Saturation of slow flows can lead to misinterpretation of vessel caliber or to misdiagnosis of stenosis or thrombosis. Artifacts due to calcification, stents, or other metallic medical devices also have to be taken into account.

A positive countereffect of this technical limitation is the ability of TOF imaging to indirectly show slow flows that contrast-enhanced imaging could not depict correctly.

Other disadvantages come from the 2D acquisition, with the well-known stair-step artifact due to nonisotropic voxels, and eventual slice incorrect registration due to patient motion.

Even considering the wide acceptance and clinical usage of TOF sequences for extracranial venous drainage study (also over 2D and/or 3D PC imaging which is not convincing and thus not commonly used in the extracranial region) [8, 11, 12, 28], SNRs (signal-to-noise ratios) and CNRs (contrast-to-noise ratios) have been proven to be inferior to those of the CE techniques [29]. This is in line with previous studies' findings of overall superiority of contrast-enhanced techniques over TOF MRV in the visualization of the cerebral venous system [5, 30, 31].

### 3.3. Extracranial 2D Cine Phase Contrast MRV Flow Quantification

**Figure 3:**
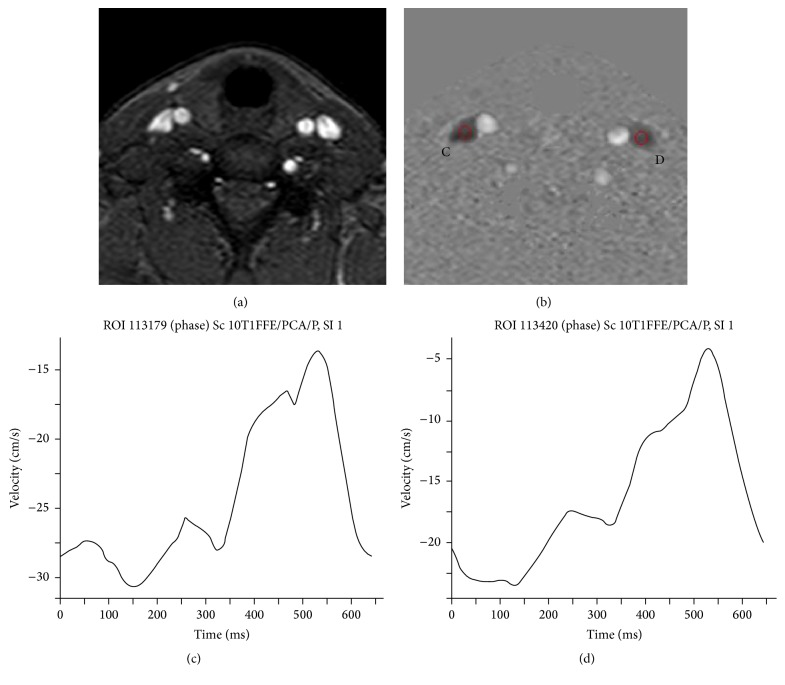
2D Cine phase contrast at C5-C6 level: (a) magnitude image that displays carotids and IJVs; (b) phase image at the same level displaying dark flow directed to the heart and bright flow directed to the brain, with C and D indicating ROIs for flow measurements within IJVs bilaterally; and (c, d) velocity curves, respectively, for C and D ROIs.

2D Cine PC sequences are commonly used to assess flow dynamics in arterial and venous vessels: in the case of jugular veins, flow quantification is an additional tool available to support the primarily structural information provided by 2D TOF MRV and if available, contrast-enhanced MRV. This enables assessment of flow-related characteristics that are otherwise commonly obtained by DS (Figure 3).

To execute a 2D Cine PC study, a pulse trigger is positioned on the subject's (left/right) index finger or, preferably, cardiac gating is used to obtain the most accurate detection of flow characteristics along cardiac cycle.

A slice of interest is acquired perpendicular to the vessel long axis (thus to flow direction) and a flow velocity curve is derived as a function of time. Flow rate can be calculated given the vessel section for that selected slice, which can be assessed through 2D TOF or contrast-enhanced imaging. Neck scout localizer or even extracranial 2D TOF MRV can be used to correctly set axial slices.

Slices can be collected at various levels, though C2-C3, C5-C6, and C7-T1 levels are the most common. VENC is generally recommended at 50 cm/s to best measure flow in the major draining veins while still having enough SNR in slow-flow vessels. Our recommendation is to acquire at least two different levels, preferably C2-C3 and C5-C6, to achieve an acceptable overview of flow characteristics and to reduce misdiagnoses [8, 11, 32, 33], avoiding unnecessarily long scanning acquisitions.

The C7-T1 level permits the study of flow characteristics of the IJV axes just above confluence with the subclavian vein, although technical difficulties are often reported for flow calculations at this level because of turbulent flow patterns at the confluence [8, 11]. If needed, extra levels can be added to the Cine PC study to identify and/or to confirm the presence of abnormal valves or wall abnormalities that may be reported by DS.

The C2-C3 level permits an assessment of downward flow from the sigmoid sinus and inferior petrosal sinus, which is in the uppermost portion of the extracranial region. The C5-C6 level can be considered as optimal to best display flow within the IJV system, combined with collateral drainage from the common facial vein and other major tributaries below the midneck level. A good level for this window is considered cranial to IJV valve and caudal to the carotid bifurcation [8, 11].

The advantage of this sequence lies in its ability to quantify the major venous flows. It can detect the duration and magnitude of any retrograde flow phenomena, as well as providing collaterally useful information on parallel arterial flows in the carotids.

In recent years, 4D flow quantification has been developed as a promising tool for representation of flow dynamics throughout the whole cardiac cycle, allowing measurement of pressure gradients along vessels [34, 35]. This technique, which currently requires long acquisition and processing times, is probably going to have a great impact on vascular flow studies. At the present moment, however, 2D Cine phase contrast still represents a valuable tool for flow measurements that can reliably be used along with ultrasound studies.

### 3.4. Contrast-Enhanced Time-Resolved MRV

Contrast-enhanced MR angiography (CE MRA) is based on T1 shortening of blood due to intravenous gadolinium chelate agent administration. This method has a considerably faster scan time, when compared to non-contrast-enhanced sequences. Generally, high temporal and spatial resolution is easily achieved, with nearly isotropic acquisitions made possible by 3D acquisitions.

Disadvantages of contrast-enhanced sequences, apart from those related to gadolinium administration common to all sequences that employ contrast, may be linked to artifacts created by signal loss due to high gadolinium chelate concentrations, in which cases T2^*∗*^ effects can dominate.

Conventional 3D contrast-enhanced MR angiography (MRA), whether in arterial or venous phase, is obtained at a single point in time after intravenous injection of paramagnetic contrast medium. Time-resolved MRA sequences (known under acronyms such as 4D-TRAK for Philips, TRICKS for GE, and TWIST for Siemens) provide a series of images that clearly display the passage of a contrast bolus first in arterial vessels and, subsequently, in venous ones [29, 30, 36–38].

Typically, a time-resolved MRA sequence includes twenty or more images that are obtained at rates as rapid as 1-2 frames per second.

**Figure 4:**
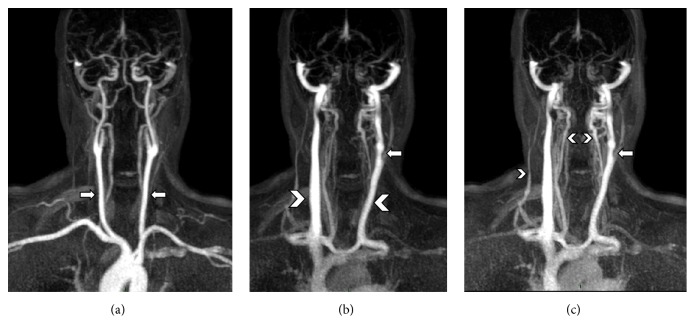
Time-resolved MRV (4D-TRAK), reconstructed as coronal MIP projections at different time points, showing (a) arterial phase with carotid axes (arrows), (b) venous phase with visualization of IJVs (arrow heads), with a segmentary stenosis of left IJV (arrow), and (c) a delayed venous stage with better visualization of venous drainage of right external jugular vein and vertebral veins (arrow heads) and a confirmation of the left IJV stenosis from the previous phase (arrow).

The basic methods for time-resolved MRA imaging are the same as those for conventional MRA sequences. A fast 3D T1-weighted GRE image is collected before the contrast agent administration, and then all the desired volumes are acquired when the contrast agent is located first within arterial vessels and then in venous collectors. Digital subtraction of the pre- and post-contrast injection images leaves only the enhanced signals from the desired vessels: coronal maximum intensity projections (MIPs) are commonly used for displaying datasets (Figure 4).

The first advantage of this sequence is that 4D MRA is able to provide dynamic information about blood flow in addition to the information given by static contrast-enhanced angiographic techniques [39]. Secondly, 4D MRA is preferred especially when timing of cerebral venous drainage is of strict interest due to its high temporal resolution. An example of this power is shown in the case of detecting early draining veins in vascular malformations. In this context, time-resolved MRA has recently been used to detect vascular malformations in the neck region and in the spine with promising results [40, 41].

Furthermore, different from classic MR angiographic bolus-tracking techniques, data acquisition of time-resolved MRA starts in the same moment as the contrast agent administration, thus reducing operator-dependence. Within a short time interval, this technique can display a clear and wide overview of the supra-aortic arterial and venous system separately. With the correct postprocessing selection of the arterial and venous vascular phase, it can also display the whole vascular system.

Excellent images can be obtained with substantially lower contrast doses compared to conventional high-spatial resolution CE MRA datasets using 4D MRA.

Time-resolved techniques also require simpler postprocessing compared to 3D CE MRA, so the radiographer work and total processing time are reduced (with a directly proportional reduced error risk). In this sense, operator-dependent errors are considerably reduced.

As previously reported, the major limitation of 4D-techniques is their lower special resolution, compared to 3D CE MRA [29]. This technique, however, can still be considered as a reliable contrast-enhanced technique for anatomical assessment of venous vessels of the neck.

## 4. Conclusions

In recent years, great attention has been dedicated to the study of the intra- and extracranial venous systems. MRV has gained a role of primary importance among other imaging techniques due to its well-known reliability, noninvasiveness, and wide availability of different sequences that can depict the diverse aspects of venous flow.

In our clinical experience, we realized that a practical and rapid MRV protocol could be useful to investigate any venous abnormalities that may be linked to a variety of etiologies that, according to recent literature, are progressively increasing in number. After an initial usage of this protocol in MS patients suspected of having CCSVI, we felt that this kind of approach to detect venous abnormalities could be of interest in a wider cohort of patients.

Considering the aforementioned overall superiority of contrast-enhanced sequences over 2D TOF and 3D PC imaging, we can still conclude that availability of a relatively short noncontrast protocol would be helpful in a variety of clinical cases in which contrast administration is not feasible and for follow-up over time of patients with compromised renal function.

A key limitation of MRV imaging is its lower image resolution when compared to angiography, which remains the gold standard exam, as MRV cannot evaluate in detail intraluminal pathologies related to valves, wall irregularities, membranes, and so forth and suffers from detection of a number of false-positive cases of vessel stenosis that may not be confirmed by catheter venography [13, 42].

Secondly, a relevant limitation of MRV approach to venous system study is represented by its “shot” nature, as it captures a fixed image of the venous system in a certain moment. 4D time-resolved imaging is an exception to this, as it can depict contrast medium flow from the injection through arterial vessels and then venous drainage.

The most accurate assessment of veins requires more effort than a simple one “shot” representation or a depiction of flow over arterial and then venous vessels over time from the same point of view, as provided by time-resolved MRV. Multiple views and breath and movement maneuvers are also needed to investigate characteristics and behavior of venous flows in different positions and intravenous pressure conditions, as is commonly performed in ultrasound studies. Any consideration regarding patient position and performed maneuvers poses risk to reproducibility of DS studies, which are extremely operator-dependent. In this sense, MRV imaging can play a role in reducing this intrinsic dependence.

In the most common MR scanners, data are usually collected in the supine position, which represents a remarkable difference from DS and angiography studies. Some scanners have been developed to perform an upright scan, thus widening the diagnostic potential of MRI [43, 44]. Influence of positional changes on cerebral venous drainage has been described on positional MR imaging by Niggemann et al. [45, 46].

Thirdly, MRV sequences, however, are not intended to substitute the essential contribution that T1w and T2w structural sequences can provide, especially in those cases in which a venous abnormality is related to extrinsic mass effect.

All things considered, MRV represents a valuable diagnostic tool in detecting intra- and extracranial venous drainage abnormalities that can help clinicians in directing diagnostic decisions, especially when performed in addition to or to confirm US and Doppler findings, without the need of ionizing radiation necessary for CT or the administration of gadolinium.

Recent findings suggest that additional research in venous system involvement in a number of different CNS disorders, with particular regard to inflammatory and neurodegenerative cases, may help in increasing our knowledge of their pathology and evolution.

## Figures and Tables

**(a):** 

	4D TRAK	2D PC neck
TR∖TI	4.5	10
TE	1.61	6.2
Matrix	428 × 217	120 × 84
Acquisition voxel	0.72/0.72	1.00/1.43/5.00
Reconstruction voxel	0.55/0.55/0.60	0.47/0.47/5.00
Number of slices	120	1
NSA		2
PC/VENC (cm/s)	/	50
WFS (Pix)/BW (Hz)	0.502/432.7	1.134/191.6
SENSE (PAT)	RL 2.5	/
Acquisition time (min)	0:57	2:49 (2x)

**(b):** 

	3D PC brain	2D TOF neck	2D PC neck
TR∖TI	20	17	10
TE	5.5	3.3	6.2
Matrix	232 × 220	224 × 224	120 × 84
Acquisition voxel	0.99/1.04/4.0	0.89/0.89/3	1.00/1.43/5.00
Reconstruction voxel	0.9/0.9/2.0	0.39/0.39/3	0.47/0.47/5.00
Number of slices	80	110	1
NSA	1	1	2
PC/VENC (cm/s)	15	/	50
WFS (Pix)/BW (Hz)	1.145/189.7	1.270/171.0	1.134/191.6
SENSE (PAT)	AP 1.5	/	/
Acquisition time	7:50	7:25	2:49 (2x)
